# Clinical Diagnostic Accuracy in the Management of Primary Stage 1 Cutaneous Malignant Melanoma in a Plastic Surgery Unit

**Published:** 1984-04

**Authors:** R. W. Griffiths, J. C. Briggs, R. W. Hiles

**Affiliations:** Senior Registrar in Plastic Surgery, Department of Plastic and Jaw Surgery, Frenchay Hospital, Bristol; Consultant Histopathologist, Department of Pathology, Frenchay Hospital, Bristol; Consultant Plastic Surgeon, Department of Plastic and Jaw Surgery, Frenchay Hospital, Bristol

## Abstract

In a retrospective review of 614 primary Stage I cutaneous malignant melanomata and 40 nonmelanoma lesions, the diagnostic accuracy(DA) for malignant melanoma was 86.2%. A positive preoperative clinical diagnosis of malignant melanoma was confirmed histologically in 564/604 (93.3%) of lesions. For 614 histologically proven malignant melanomata a correct pre-operative clinical diagnosis had been made in 564/614 (91.9%).

An additional 172 patients were referred for wider excisional surgery within 3 months of a biopsy elsewhere. For the total 786 (614+172) patients, the incidence of biopsy of a clinicially unsuspected (but subsequently histologically proven) malignant melanoma prior to referral to the Plastic Surgery Unit was lowest for lesions of the head and neck (18.3%) and lower limb (19.0%), and highest (almost half of the patients) for lesions of the hands and fingers.

Previous reports of the poor level of clinical diagnostic accuracy in cutaneous malignant melanoma have not been confirmed in the present study.


### Clinical Diagnostic Accuracy in the Management of Primary Stage I Cutaneous Malignant Melanoma in a Plastic Surgery Unit*

**R. W. Griffiths, M.S., F.R.C.S.**
Senior Registrar in Plastic Surgery, Department of Plastic and Jaw Surgery, Frenchay Hospital, Bristol
**J. C. Briggs, F.R.C.Path.**
Consultant Histopathologist, Department of Pathology, Frenchay Hospital, Bristol
**R. W. Hiles, F.R.C.S., F.R.C.S. Ed.**
Consultant Plastic Surgeon, Department of Plastic and Jaw Surgery, Frenchay Hospital, Bristol

### SUMMARY

In a retrospective review of 614 primary Stage I cutaneous malignant melanomata and 40 non-melanoma lesions, the diagnostic accuracy(DA) for malignant melanoma was 86.2%. A positive pre-operative clinical diagnosis of malignant melanoma was confirmed histologically in 564/604 (93.3%) of lesions. For 614 histologically proven malignant melanomata a correct pre-operative clinical diagnosis had been made in 564/614 (91.9%).

An additional 172 patients were referred for wider excisional surgery within 3 months of a biopsy elsewhere. For the total 786 (614+172) patients, the incidence of biopsy of a clinicially unsuspected (but subsequently histologically proven) malignant melanoma prior to referral to the Plastic Surgery Unit was lowest for lesions of the head and neck (18.3%) and lower limb (19.0%), and highest (almost half of the patients) for lesions of the hands and fingers.

Previous reports of the poor level of clinical diagnostic accuracy in cutaneous malignant melanoma have not been confirmed in the present study.

### INTRODUCTION

It has been suggested that the clinical diagnosis of cutaneous malignant melanoma poses few problems for the experienced clinician.[1] However others[2,3] have emphasised the very variable nature of biopsy techniques currently used on lesions which are later shown histologically to be malignant melanomata, and have highlighted the particular difficulties which inadequate biopsy material creates for the histopathologist in his attempt to correctly microstage the tumour by the methods of Clark[4] and Breslow.[5] In many series, predominantly from non surgical departments, the reported clinical diagnostic accuracy rate is poor.[6-9] We therefore sought to define the various pathways to diagnosis for a large number of patients with primary cutaneous malignant melanoma presenting to one surgical unit where malignant melanomata represent 10% of all skin tumours excised annually. The implications of various diagnostic approaches for definitive surgical management of cutaneous malignant melanoma are considered.

### MATERIALS AND METHODS

All consecutive admissions for cutaneous malignant melanoma* to the Plastic Surgery Unit at Frenchay Hospital were identified for the 12 year period January 1967–December 1978 inclusive. Patients were identified from four sources:

(1) Inpatient and outpatient operation registers.
(2) Hospital Activity Analysis (HAA) computer record of discharges after treatment for malignant melanoma.

* Based on a paper read to the Surgical Club of South West England Autumn Meeting 1983, Weston-super-Mare, Friday 21st October 1983.

* Including cases of lentigo maligna and lentigo maligna melanoma.

*Address for correspondence:* R. W. Griffiths, M.S., F.R.C.S., Senior Registrar in Plastic Surgery, Department of Plastic and Jaw Surgery, Frenchay Hospital, Bristol BS16 1LE.

(3) Records of the Malignant Melanoma Registry, Frenchay Hospital.
(4) Records of the Department of Histopathology, Frenchay Hospital.

The clinical notes or microfilm records of all patients thought (from one or more of the above sources) to have been treated for malignant melanoma were studied by one worker (R. W. G.). The use of the operation registers enabled patients with a presumptive diagnosis of malignant melanoma to be identified. Patients identified from the HAA computer record as being treated exclusively by other units within the hospital were excluded from review.

The surgical treatment policy throughout the period under review was standard. When a firm clinical diagnosis of malignant melanoma was made after naked eye inspection of the lesion, then primary definitive surgery by wide margin excision (WE) of the lesion with either direct closure or more usually skin grafting was performed. Margins of normal tissue (skin and fat) resected around the tumour varied from 5–10 cm on the trunk, to 5 cm on the limbs, to margins of 1 cm or less on the head and neck (where anatomical constraints limited resection margins). Resections were carried out to the level of, but not including deep fascia. If there was doubt over the clinical diagnosis then either minimal margin excision biopsy (MMEB) with urgent paraffin section, or MMEB with immediate frozen section histological examination were performed. Definitive surgery proceeded on the same day if frozen section and clinical appearance both supported the diagnosis of malignant melanoma. There was a longer delay to definitive surgery when frozen section examination was inconclusive and paraffin section examination followed.

Prophylactic lymph node dissections were not performed. When patients were referred after biopsy procedures elsewhere, wide margin excision of the biopsy site was performed as for primary tumours. During the 12 year review period the pre-operative clinical assessment of these tumours was performed by six consultant plastic surgeons and approximately eight senior registrars/registrars.

### RESULTS

In the 12 year period 1967–78 a total of 1002 patients were treated on the Plastic Surgery Unit at Frenchay Hospital for various stages of cutaneous malignant melanoma.

A total of 154/1002 (15.4%) were treated for either recurrent or metastatic disease (primary tumour treated prior to 1967), or were referred for definitive surgery later than 3 months after the tumour had been biopsied elsewhere. A further 62 patients presented not only with the primary lesion, but also with either satellite lesions, in transit metastases or enlarged regional lymph nodes. These 216 (154+62) patients are not considered further here.

786 patients presented with clinical Stage I disease, of which 614 were treated exclusively by the Plastic Surgery Unit and a further 172 were treated by WE within 3 monhs of an initial biopsy procedure elsewhere (Table 1). 614 patients therefore were treated primarily by the Plastic Surgery Unit in whom there were no indications *either* from previous biopsy *or* from the presence of metastatic disease that the lesion was a malignant melanoma, other than the clinical appearance of the primary tumour (Table 2). In 50/614 (8.1%) the clinical diagnosis was in doubt and a biopsy procedure was first performed. A total of 27/614 (4.4%) were submitted to MMEB and

**Table 1**

**Cutaneous malignant melanoma, Plastic Surgery Unit, Frenchay Hospital 1967–78**

*Patients with Clinical Stage I disease*

| *Site* | *Total* | *Treated primarily by the Plastic Surgery Unit* | *Treated by the Plastic Surgery Unit within 3 months of a biopsy procedure elsewhere* |
|---|---|---|---|
| Lower limb | 310 | 251 | 59 (19.0%) |
| Head and neck | 208 | 170 | 38 (18.3%) |
| Trunk | 128 | 101 | 27 (21.1%) |
| Upper limb | 85 | 57 | 28 |
| Feet and toes | 40 | 27 | 13 |
| Hands and fingers | 15 | 8 | 7 |
| Total | 786 | 614 (78.1%) | 172 (21.9%) |

**Table 2**

**Cutaneous malignant melanoma, Plastic Surgery Unit, Frenchay Hospital 1967–78**

*Patients with Stage I disease treated primarily by the Plastic Surgery Unit*

| *Site* | *Total* | *Primary wide excision (WE) with no initial biopsy* | *Initial biopsy and paraffin section* | *Initial frozen section followed by paraffin section* |
|---|---|---|---|---|
| Lower limb | 251 | 229 (91.3%) | 12 | 10 |
| Head and neck | 170 | 168 (98.8%) | 0 | 2 |
| Trunk | 101 | 93 (92.1%) | 2 | 6 |
| Upper limb | 57 | 44 | 8 | 5 |
| Feet and toes | 27 | 22 | 1 | 4 |
| Hands and fingers | 8 | 8 | 0 | 0 |
| Total | 614 | 564 (91.9%) | 23 (3.7%) | 27 (4.4%) |

immediate frozen section histological examination. The diagnosis of a naevocytic/melanocytic lesion was confirmed immediately in 19 patients and on the basis of this coupled with the clinical appearance an immediate further wide excision of surrounding skin and subcutaneous fat was performed. Subsequent paraffin section examination confirmed malignant melanoma for all 19 lesions. In 8/27 the frozen section appearance and the clinical picture were not sufficiently conclusive to warrant immediate further wide excision and in consequence further excisional surgery was delayed 2–27 days (mean 9 days) until malignant melanoma was confirmed by paraffin section examination. (The delay was less than 8 days in 6 of these 8 patients.) (Frozen section examination in a further 7 patients diagnosed benign lesions for whom MMEB was the definitive treatment, in all 7 paraffin section confirmed frozen section diagnosis.) MMEB, urgent paraffin section followed by further wide excision and skin grafting were performed in 23/614 (3.7%) patients. For these patients the delay from biopsy to definitive surgical excision ranged 3–90 days (mean 18 days). (The delay was less than 30 days in 20 of these 23 patients.)

Of the 614 histologically proven malignant melanomata a firm pre-operative diagnosis had been made in 564/614 (91.9%) and these had been treated by initial wide margin excision without prior biopsy (Table 2).

During the 12 year period 40 patients were submitted to WE of skin lesions thought clinically to be undoubted malignant melanomata, but which were shown histologically not to be (Tables 3–5). Of the 30 patients treated by wide excision and split thickness skin grafting, only 6 could have been managed by direct wound closure even if MMEB had been employed (either because of the size of the lesion or its anatomical site). If the toe amputation is also

**Table 3**

**Lesions excised as presumptive malignant melanomata**

| *Site of lesions* | |
|---|---|
| Lower limb | 14 |
| Trunk | 12 |
| Head and neck | 8 |
| Upper limb | 6 |
| Total | 40 |

**Table 4**

**Lesions excised as presumptive malignant melanomata**

| *Histopathological diagnoses* | |
|---|---|
| Seborrhoeic keratosis/wart | 8 |
| Haemangioma | 7 |
| Basal cell carcinoma (with or without pigmentation) | 6 |
| Benign cellular naevus | 5 |
| Histiocytoma | 4 |
| Benign lentigo/benign melanoma | 4 |
| Active junctional naevus | 1 |
| Pigmented papilloma | 1 |
| Squamous cell carcinoma | 1 |
| Reticulosarcoma | 1 |
| Epidermoid cyst | 1 |
| Psoriasis | 1 |
| Total | 40 |

**Table 5**

**Lesions excised as presumptive malignant melanomata**

| *Types of surgical procedure performed* | |
|---|---|
| Wide excision and split thickness skin grafting | 30 |
| Wide excision and direct closure | 7 |
| Wide excision and full thickness skin grafting | 1 |
| Wide excision and rotation skin flap | 1 |
| Amputation of second toe through the metatarsophalangeal joint | 1 |
| Total | 40 |

included it can be expressed that 7/604 (1.2%) of patients might have been spared an unnecessarily extensive surgical procedure if an initial MMEB had been performed. Thus of all primary wide excisions (564 melanomata and 40 non-melanoma lesions) performed on the basis of the clinical diagnosis alone of malignant melanoma, 40/604 (6.6%) proved to be for lesions other than malignant melanoma.

A positive pre-operative clinical diagnosis of malignant melanoma was therefore confirmed histologically for 564/604 (93.3%) of lesions.

Expressing these data in terms of diagnostic accuracy (DA)[9,10], the DA was 86.2%.*

When tumours were biopsied prior to referral to the Plastic Surgery Unit, biopsy was most common for lesions of hands/fingers and feet/toes (more than 1/3rd of lesions), and least common (less than 20%) for lesions of the head and neck or lower limbs (Table 1).

### DISCUSSION

Although the Plastic Surgery Unit at Frenchay Hospital has long served as a referral centre for cutaneous malignant melanoma, it also treats large numbers of patients with other skin neoplasms (benign and malignant). Therefore many patients are referred to the Unit without any previous specialist diagnosis.

$$* \, DA(\%) = \frac{(A) \times 100}{(A)+(B)+(C)} = \frac{564 \times 100}{564+40+50} = 86.2\%.$$

A = tumours diagnosed clinically as malignant melanoma and confirmed as such histologically.

B = tumours diagnosed clinically as malignant melanoma but shown histologically not to be malignant melanoma.

C = tumours not clearly diagnosed clinically as malignant melanoma e.g.) excision biopsies with frozen or urgent paraffin section, but shown histologically to be malignant melanoma.

The level of diagnostic accuracy (86.2%) determined from this study approaches the figure of over 90% which Clark[11] has suggested should be achieved, and far exceeds figures for DA determined for previously published series of small numbers of patients with proven malignant melanoma (Table 6). This high level of clinical diagnostic accuracy must be considered against the background of the specific surgical policy in force during the review period. It seems likely that, conscious of the large size of defect they will create, surgeons may be particularly critical in their clinical assessment of skin lesions which may be potential melanomata.

**Table 6**

**Diagnostic accuracy rates derived from published series**

| *Authors* | *Number of lesions with a correct positive pre-operative diagnosis of malignant melanoma confirmed histologically* | *Diagnostic accuracy (%)* |
|---|---|---|
| Ewing and Powell (1951) | 14 | 46.7% |
| Swerdlow (1952) | 16 | 23.5% |
| Becker (1954) | 72 | 29.0% |
| McMullan and Hubener (1956) | 44 | 27.8% |
| Kopf et al (1975) | 76 | 64.4% |
| Present series | 564 | 86.2% |

Despite the otherwise poor levels of diagnostic accuracy in most reports,[6–9,12,13] MacKie has reported an 84% accuracy of positive pre-operative diagnosis when all lesions were examined clinically with skin microscopy.[14] The comparable figure for naked eye assessment (by some 14 surgeons) in the present series is 93.3%.

In the present series of all cases of histologically proven cutaneous Stage I malignant melanoma treated primarily by the Plastic Surgery Unit 91.9% were managed by initial wide excision on the basis of clinical appearance alone, without prior biopsy of any sort. A further 40 lesions were similarly excised widely but proved not to be melanoma (Tables 4 and 5). These 40 patients represent 6.6% of all patients submitted to wide excision of their tumour on the basis of clinical appearance alone. Although advocates of immediate frozen section histological examination would suggest that this technique should avoid such unnecessarily wide excisions, it is of interest to note that one of the most enthusiastic reports of routine use of frozen section in the management of suspected malignant melanoma,[15] indicated that of 82 wide excisions performed prior to the result of frozen section examination (because of confidence in the clinical diagnosis of malignant melanoma), 16 (19.5%) proved to be for non melanoma lesions. Rather than being employed routinely, frozen section is likely to be most valuable in distinguishing melanocytic from non-melanocytic lesions when doubt exists clinically,[16] but it may prove very difficult with this technique to differentiate benign from malignant naevocytic lesions at the dermo-epidermal junction.[17] Frozen section techniques will also pose diagnostic problems in lesions exhibiting regression, and frozen section specimens are likely to be thicker than comparable paraffin section specimens.[18] Therefore when reports of maximal tumour thickness are given, it should be made clear whether these measurements have been made on frozen or paraffin section preparations. This has become particularly important since the adoption of routine measurement of maximal tumour thickness in the histological assessment of primary malignant melanoma.[5]

Although we do not know how various biopsy techniques (incisional, shave, punch etc) affect prognosis in malignant melanoma,[19,20] the most compelling reason for avoiding inadequate biopsy methods is the tissue distortion that these may cause. The histopathologist can only accurately microstage the tumour (at present most importantly by measuring the maximal thickness) if he is presented with the whole lesion surrounded by an adequate 'cuff' of adjacent normal tissue to which the tumour may be related. Even such narrow margin excision biopsy may sometimes create a defect which cannot be sutured directly and may require skin grafting. This is likely to be particularly true for lesions of feet/toes and hands/fingers where there is minimal tissue laxity; in the present series 20/55 (over 1/3rd) of lesions in these sites were biopsied prior to referral for definitive surgery. A higher index of suspicion for the diagnosis of malignant melanoma in these peripheral sites is clearly needed. Compromise of the adequacy of such tumour excision biopsy in these areas could be avoided by referral to a surgical unit.

Although this study relates to data for a period when wide margin excision of all suspected melanoma lesions was the policy, it should be emphasised that this report should not be construed as advocating such wide margin excision as definitive treatment for cutaneous malignant melanoma now. The optimal resection margins (to limit the incidence of local tumour recurrence) have yet to be determined, and recently several groups of workers have questioned conventional excision margins and have variously suggested reduction in clearance margins.[21-23]

With the rising incidence of cutaneous malignant melanoma in this country,[24] all clinicians should familiarise themselves with the irregularities of colour, surface contour and margin which are the hallmarks of the tumour.[25]

High levels of diagnostic accuracy in the management of malignant melanoma of the skin need not be restricted to Plastic Surgery Units, but clearly referral centres dealing with large numbers of cases have the advantage of experience.

### ACKNOWLEDGEMENTS

We are grateful to all consultants Mr. G. M. Fitzgibbon, Mr. D. C. Bodenham, Mr. R. T. Routledge, Mr. R. W. Pigott and Mr. P. L. G. Townsend for allowing us to review patients admitted under their care. We are particularly indebted to Mr. D. C. Bodenham who developed the interest for melanoma care at this unit. We also thank Mrs. Jeanne Bayley (Melanoma Registry Secretary) for many hours of work in tracing notes and cross-referencing.

### REFERENCES

1. DU VIVIER, A. (1982) Spotting the malignant melanoma. *Br.Med.J.* **285**, 670–671.
2. ROSES, D. F., ACKERMAN, A. B., HARRIS, M. N., WEINHOUSE, G. R. and GUMPORT, S. L. (1979) Assessment of biopsy techniques and histopathological interpretations of primary cutaneous malignant melanoma. *Ann.Surg.* **189**, 294–297.
3. MACY-ROBERTS, E. and ACKERMAN, A. B. (1982) A critique of techniques for biopsy of clinically suspected malignant melanomas. *Am.J.Dermatopathol.* **4**, 391–398.
4. CLARK, W. H. FROM, L., BERNARDINO, E. H., and MIHM, M. C. (1969) The histogenesis and biologic behaviour of primary human malignant melanoma of the skin. *Cancer Res.* **29**, 705–726.
5. BRESLOW, A. (1970) Thickness, cross sectional area and depth of invasion in the prognosis of cutaneous melanoma. *Ann.Surg.* **172**, 902–908.
6. BECKER, S. W. (1954) Pitfalls in the diagnosis and treatment of melanoma. *Arch.Dermatol.Syphilol.* **69**, 11–30.
7. SWERDLOW, M. (1952) Nevi: a problem of misdiagnosis. *Am.J.Clin.Path.* **22**, 1054–1060.
8. EPSTEIN, E., BRAGG, K., and LINDEN, G. (1969) Biopsy and prognosis of malignant melanoma. *J.A.M.A.* **208**, 1369–1371.
9. KOPF, A. W., MINTZIS, M., and BART, R. S. (1975) Diagnostic accuracy in malignant melanoma. *Arch.Dermatol.* **111**, 1291–1292.

10. LIGHTSTONE, A. C., KOPF, A. W., and GARFINKEL, L. (1965) Diagnostic accuracy: a new approach to its evaluation. *Arch.Dermatol.* **91**, 497–502.
11. CLARK, W. H. (1976) Clinical diagnosis of cutaneous malignant melanoma. *J.A.M.A.* **236**, 484–485.
12. EWING, M. R., and POWELL, T. (1951) Some observations on the diagnosis of clincially pigmented skin tumours. *Br.J.Surg.* **38**, 442–454.
13. McMULLAN, F. H. and HUBENER, L. F. (1956) Malignant melanoma: a statistical review of clinical and histological diagnoses. *Arch.Dermatol.* **74**, 618–619.
14. MacKIE, R. M. (1971) An aid to the preoperative assessment of pigmented lesions of the skin. *Br.J.Dermatol* **85**, 232–238.
15. LITTLE, J. H. and DAVIS, N. C. (1974) Frozen section diagnosis of suspected malignant melanoma of the skin. *Cancer* **34**, 1163–1172.
16. HUGHES, L. E. (1975) The place of frozen section in the practical management of melanoma. *Br.J.Surg.* **62**, 840–844.
17. MILTON, G. W. and JELIHOVSKY, T. (1962) Frozen section examination in the diagnosis of cutaneous malignant melanoma (melanoblastoma). *Med.J.Aust.* **2**, 503–504.
18. SHAFIR, R., HISS, J., TSUR, H. and BUBIS, J. J. (1983) Pitfalls in frozen section diagnosis of malignant melanoma. *Cancer* **51**, 1168–1170.
19. RAMPEN, F. H. J., VAN HOUTON, W. A. and HOP, W. C. J. (1980) Incisional procedures and prognosis in malignant melanoma. *Clin.Exp.Dermatol.* **5**, 313–320.
20. BAGLEY, F. H., CADY, B., LEE, A. and LEGG, M. A. (1981) Changes in clinical presentation and management of malignant melanoma. *Cancer* **47**, 2126–2134.
21. CASCINELLI, N., VAN DER ESCH, E. P., BRESLOW, A., MORABITO, A. and BUFALINO, R. (1980) Stage I melanoma of the skin; the problem of resection margins. *Eur.J.Cancer.Clin.Oncol.* **16**, 1079–1085.
22. DAY, C. L., MIHM, M. C., SOBER, A. J., FITZPATRICK, T. B. and MALT, R. A. (1982) Narrower margins for clinical Stage I malignant melanoma *N.Eng.J.Med.* **306**, 479–482.
23. ELDER, D. E., GUERRY, DU P., HEIBERGER, R. M. et al. (1983) Optimal resection margin for cutaneous malignant melanoma. *Plast.Reconstr.Surg.* **71**, 66–78.
24. SORAHAN, T. (1982) Incidence of malignant melanoma in England and Wales 1968–1977. *Lancet* **2**, 562.
25. MIHM, M. C., FITZPATRICK, T. B., LANE BROWN, M. M., RAKER, J. W., MALT, R. A. and KAISER, J. S. (1973) Early detection of primary cutaneous malignant melanoma. A colour atlas. *N.Eng.J.Med.* **289**, 989–996.

